# Human Immunodeficiency Virus-1 Uses the Mannose-6-Phosphate Receptor to Cross the Blood-Brain Barrier

**DOI:** 10.1371/journal.pone.0039565

**Published:** 2012-06-25

**Authors:** Shinya Dohgu, Jan S. Ryerse, Sandra M. Robinson, William A. Banks

**Affiliations:** 1 Department of Pharmaceutical Care and Health Sciences, Faculty of Pharmaceutical Sciences, Fukuoka University, Fukuoka, Japan; 2 Department of Pathology, Saint Louis University Health Sciences Center, St. Louis, Missouri, United States of America; 3 Division of Geriatric Medicine, Department of Internal Medicine, Saint Louis University School of Medicine, St. Louis, Missouri, United States of America; 4 Geriatric Research Educational and Clinical Center-Veterans Affairs Puget Sound Health Care System, Seattle, Washington, United States of America; 5 Division of Gerontology and Geriatric Medicine, Department of Medicine, University of Washington, Seattle, Washington, United States of America; Biological Research Centre of the Hungarian Academy of Sciences, Hungary

## Abstract

HIV-1 circulates both as free virus and within immune cells, with the level of free virus being predictive of clinical course. Both forms of HIV-1 cross the blood-brain barrier (BBB) and much progress has been made in understanding the mechanisms by which infected immune cells cross the blood-brain barrier BBB. How HIV-1 as free virus crosses the BBB is less clear as brain endothelial cells are CD4 and galactosylceramide negative. Here, we found that HIV-1 can use the mannose-6 phosphate receptor (M6PR) to cross the BBB. Brain perfusion studies showed that HIV-1 crossed the BBB of all brain regions consistent with the uniform distribution of M6PR. Ultrastructural studies showed HIV-1 crossed by a transcytotic pathway consistent with transport by M6PR. An *in vitro* model of the BBB was used to show that transport of HIV-1 was inhibited by mannose, mannan, and mannose-6 phosphate and that enzymatic removal of high mannose oligosaccharide residues from HIV-1 reduced transport. Wheatgerm agglutinin and protamine sulfate, substances known to greatly increase transcytosis of HIV-1 across the BBB *in vivo*, were shown to be active in the *in vitro* model and to act through a mannose-dependent mechanism. Transport was also cAMP and calcium-dependent, the latter suggesting that the cation-dependent member of the M6PR family mediates HIV-1 transport across the BBB. We conclude that M6PR is an important receptor used by HIV-1 to cross the BBB.

## Introduction

Human immunodeficiency virus-1 [1] crosses the blood-brain barrier (BBB) to infect the central nervous system (CNS). It crosses the BBB both within infected immune cells and as free virus [2], [3], [4]. How free HIV-1 crosses the BBB is unclear as the brain endothelial cells that comprise the vascular BBB are both CD4 and galactosylceramide negative [5]. In vivo and in vitro studies show that interactions between the HIV-1 viral coat glycoprotein gp120 and unknown glycoproteins on the cell membranes of brain endothelia interact to induce a type of vesicular transport termed adsorptive transcytosis [4], [6], [7], a transport that is enhanced by protamine sulfate, wheatgerm agglutinin, and heparan sulfate, all of which likely act by binding to the unknown glycoproteins [7], [8], [9].

Evidence suggests that high mannose type glycans are involved in HIV-1 internalization. High mannose glycans are one type of carbohydrate moiety found on gp120 [10]. By way of these mannose residues, gp120 can bind to and be internalized by macrophages through a CD4-independent, mannose-specific endocytic receptor pathway [11]; this is consistent with the high mannose sites being related to HIV-1 infectivity [12].

One such mannose binding receptor is the insulin-like growth factor-II/mannose-6 phosphate receptor (M6PR). This multi-ligand transporter uniquely binds phosphorylated mannose (M6P), a feature it uses to identify and route lysosomal enzymes to the lysosomal compartment. HIV-1 is also routed to lysosomes by an unknown endocytic process that is independent of CD4 [13], [14]. Mannose-6 phosphate can inhibit gp160 binding [15], suggesting that gp160 binds to the M6PR; furthermore, microglial uptake of HIV-1 involves M6PR [16]. The M6PR is involved in the trafficking of at least one other virus: herpes varicella-zoster [17]. The M6PR has recently been shown to be an inducible transporter at the BBB for lysosomal enzymes [18], [19], [20]. These various lines of evidence led us to postulate that the M6PR could be involved in the transport of HIV-1 across the BBB.

## Methods

### Radioactive labeling of HIV-1 and bovine albumin

The virus HIV-1 (MN) CL4/CEMX174 (T1) prepared and rendered noninfective by aldrithiol-2 treatment as previously described [21], [22] was a kind gift of the National Cancer Institute, NIH. In brief, HIV-1(MN) CL.4/CEMX174(T1) was prepared by infecting CEMX174(T1) cells (obtained from Peter Cresswell at the Howard Hughes Medical Center) using virus obtained from the HIV-1(MN)/H9CL.4 cell line [23]. HIV-1(MN) CL.4/CEMX174(T1) cell cultures were grown in suspension using 850 cm2 roller bottles. Cells were maintained in complete RPMI 1640 supplemented with 10% heat-inactivated fetal bovine serum, 100 U/ml penicillin, and 100 ug/ml streptomycin. Each roller bottle contained 300 ml of culture and was incubated at 37°C while rotating at 0.20 rpm on roller racks. Cultures were harvested at 3 to 4 day intervals by decanting 20 ml from each roller bottle and refeeding with 20 ml of fresh medium. Immediately after harvesting, the material was filtered using a Prostack filtration system (Millipore Corp) containing a 0.8 A membrane. The filtrate was treated with a final concentration of 1mM aldrithiol-2 and stored overnight at 4°C while stirring. Virus from infected cells was recovered by continuous flow ultracentrifugation using a CF-32 Ti rotor (Beckman Instruments) containing a 280 ml sucrose gradient (25–50%; RNAse free) in TNE buffer (0.01MTris–HCl pH 7.40, 0.1MNaCl, and 1mMEDTA in Milli-Q water). After centrifugation, the gradient was monitored for absorbance at 280 nm and collected in 25 ml fractions. The density of each fraction was determined with a refractometer (Bausch and Lomb). Virus containing fractions were identified by UV absorption and density. Peak fractions were pooled, diluted to below 20% sucrose with TNE buffer, ultracentrifuged to a pellet, and resuspended in TNE at 1000x relative to the starting cell culture filtrate. Aliquots were stored in a liquid N2 vapor phase freezer.

The virus was radioactively labeled by the chloramine-T method, a method which preserves vial coat glycoprotein activity [24], [25]. Two mCi of ^131^I-Na (Perkin Elmer, Boston, MA), 10 ug of chloramine-T (Sigma Chemical Co, St. Louis, MO), and 5.0 ug of the virus were incubated together for 60 sec. The radioactively labeled virus (I-HIV) was separated from free iodine on a column of Sepharose 200-B (Sigma) and had a specific activity estimated to be between 50–150 Ci/g. Bovine serum albumin (Sigma; 5 ug) was labeled by the chloramine-T method with 0.5 mCi of ^125^I-Na (Perkin Elmer) and purified on a G-10 column (I-Alb).

### Brain uptake of I-HIV-1

Brain uptake of I-HIV was determined by the brain perfusion method of Banks et al. [26]. Male CD-1 mice from our in-house colony (VA St. Louis, MO) were anesthetized with an ip injection of 0.2 ml urethane (40%). All animal studies were approved by the IACUC at the John Cochran Veterans Affairs Medical Center in St. Louis, MO and were conducted at that facility. I-HIV was diluted to a concentration of 1×(10^5^) cpm/ml in Zlokovic's buffer (pH 7.4; 7.19 g/l NaCl, 0.3 g/l KCl, 0.28 g/l CaCl_2_, 2.1 g/l NaHCO_3_, 0.16 g/l KH_2_PO_4_, 0.17 g/l anhydrous MgCl_2_, 0.99 g/l D-glucose, and 10 g/l bovine serum albumin (BSA; which was added on the day of perfusion). The thorax was opened, the heart was exposed, the descending thoracic aorta was clamped, and both jugular veins were severed. A 26-gauge butterfly needle was inserted into the left ventricle of the heart, and the buffer containing the I-HIV was infused at a rate of 2 ml/min for 2, 4, 6, 8, or 10 min (n = 6/time point). At the end of the perfusion time, 20 ml of lactated Ringer's solution was infused through the heart cannula in less than one minute to wash out the vascular space of the brain and any virus loosely adhering to the luminal surface of the brain vasculature. Mice were then decapitated and the brain was collected, dissected into 10 regions (frontal cortex, parietal cortex, occipital cortex, striatum, hippocampus, hypothalamus, thalamus, cerebellum, midbrain, pons-medulla) after the method of Glowinski and Iversen [27], and each region weighed and counted in a gamma counter for 3 min. The olfactory bulb was also collected, weighed, and its level of radioactivity determined. Whole brain values were determined by summing the weights and radioactivity levels of the constituent 10 brain regions for that brain without inclusion of olfactory bulb. The brain/perfusion ratios (µl/g) for each region and for whole brain were calculated by the following formula:

Brain/perfusion ratio  =  (cpm/g of brain)/(cpm/µl of perfusion).

The pharmacokinetic parameters of unidirectional influx rate (Ki, µl/g-min) and initial volume of distribution within brain (Vi, µl/g) were determined by first plotting the brain/perfusion ratios (µl/g) against time (min) for each brain region. The Ki was taken as the slope and Vi as the Y-intercept for the linear portion of the brain/perfusion ratio vs time relation. The Ki and Vi with their error terms were calculated by the least squares method with the Prism 5.0 statistical package (GraphPad, Inc, San Diego, CA).

### HIV-1 electron microscopy

Adult male CD-1 mice aged 8–10 weeks from our in house colony (VA-St. Louis) received an injection into the jugular vein of 200 ul lactated Ringers solution containing 1% BSA with or without 20 ug of HIV-1. The mice were decapitated 5 min after the jugular injection, the brain sliced into 12 coronal sections, and the 6th slice placed in 2.5% gluteraldehyde solution (2.5% glutaraldehyde in 0.1 M sodium cacodylate buffer, pH 7.25 containing 3% sucrose and 2 mM calcium chloride). Slices were submitted for EM with the EM evaluator blinded as to which mice received HIV in their injections.

#### Tissue Processing for Standard Transmission Electron Microscopy

The cerebral cortex from the 6^th^ slice was further chopped in fixative with a razor and transferred to fresh fixative for overnight fixation at 4°C. After washing in 0.1 M sodium cacodylate buffer, pH 7.25 containing 5% sucrose, the tissue was post-fixed in 1% osmium tetroxide in 0.1 M sodium cacodylate buffer, pH 7.25 containing 2% sucrose overnight at 4°C. This was followed, at room temperature, by a 2×15 min wash in distilled water, 1 h incubation in 2% aqueous uranyl acetate, dehydration through graded ethanols to 100% ethanol, 2×15 min in propylene oxide and an overnight incubation in a 1∶1 mixture of Polybed resin and propylene oxide. The tissue was then incubated in fresh Polybed resin (PolySciences) for 6 h, transferred to BEEM capsules filled with fresh resin and polymerized overnight at 70°C. Thin sections were cut on a Reichert Ultracut E ultramicrotome, collected on 200 mesh copper grids, stained with uranyl acetate and lead citrate, and viewed and photographed with a JEOL 100CX electron microscope.

#### Immuno-Gold Cytochemistry

Tissue processing and immuno-gold labeling was carried out as previously described [28], [29]. Tissue pieces from the 6^th^ anterior-posterior brain slice were fixed with 4% paraformaldehyde and 1% glutaraldehyde in PBS, pH 7.2, overnight at 4°C. The tissue was then washed with PBS, dehydrated through graded ethanols to 100% ethanol and infiltrated overnight in LR White resin (PolySciences), all at 4°C. The tissue was then embedded in fresh LR White resin in BEEM capsules and polymerized overnight at−20°C by UV irradiation. Thin sections were collected on formvar-coated nickel grids and incubated on 20 ul drops of the following solutions in a closed humidified container at room temperature unless noted otherwise: PBS (1×1 min), 0.02M glycine (3×1 min), 1% bovine serum albumen (BSA) and 1% fish gelatin in PBS (1×10 min), 0.1% BSA in PBS (1×1 min), goat anti-human HIV-1 gp120 (HIV-1SF2 gp120 antiserum from NIH aids reagents facility) or normal goat serum (Sigma) diluted 1∶10–1∶100 in 0.1% BSA in PBS (overnight at 4°C), 0.1% BSA in PBS (4×1 min), protein A-10 nm colloidal gold diluted 1∶100 in 0.1% BSA in PBS (1 hour), PBS (1×5 min), 2.5% glutaraldehyde in PBS (1×5 min), PBS (2×5 min) and distilled water (2×5 min). After air drying the grids were post-stained with uranyl acetate and lead citrate and examined and photographed in a JEOL 100CX EM.

### Primary culture of mouse brain microvascular endothelial cells (BMECs)

BMECs were isolated by a modified method of Szabó et al. [30] and Nakagawa et al. [31]. In brief, the cerebral cortices from 8-week-old CD-1 mice were cleaned of meninges and minced. The homogenate was digested with collagenase type II (200 U/mL; Invitrogen, Carlsbad, CA) and DNase I (30 U/mL; Sigma, St. Louis, MO) in Dulbecco's modified Eagle's medium (DMEM) (Invitrogen) containing 100 units/mL penicillin, 100 µg/mL streptomycin, 50 µg/mL gentamicin and 2 mM GlutaMAX™-I (Invitrogen) at 37°C for 40 min. Neurons and glial cells were removed by centrifugation in 20% bovine serum albumin (BSA)-DMEM (1,000×g for 20 min). The microvessels obtained in the pellet were further digested with collagenase/dispase (1 mg/mL; Roche, Mannheim, Germany) and DNase I (30 U/mL) in DMEM at 37°C for 30 min. Microvessel endothelial cell clusters were separated by 33% Percoll (Amersham Biosciences, Piscataway, NJ) gradient centrifugation (1,000×g for 10 min). The obtained microvessel fragments were washed in DMEM (1,000×g for 10 min) and seeded on 60 mm culture dishes (BD FALCON™, BD Biosciences, Franklin Lakes, NJ) coated with 0.05 mg/mL fibronectin (Sigma), 0.05 mg/mL collagen I (Sigma) and 0.1 mg/mL collagen IV (Sigma). They were incubated in DMEM/Nutrient mixture F-12 HAM (DMEM/F-12) (Invitrogen) supplemented with 20% plasma derived bovine serum (PDS, Animal Technologies, Tyler, TX), 100 units/mL penicillin, 100 µg/mL streptomycin, 50 µg/mL gentamicin, 2 mM GlutaMAX™-I and 1 ng/mL basic fibroblast growth factor (bFGF; Sigma) at 37°C with a humidified atmosphere of 5% CO_2_/95% air. On the next day, the BMECs migrated from the isolated capillaries and started to form a continuous monolayer. To eliminate contaminating cells (mainly pericytes), BMECs were treated with 4 µg/mL puromycin (Sigma) for the first 2 days (Perrière et al., 2005). After 2 days of the treatment, puromycin was removed from the culture medium. Culture medium was changed every other day. After 7 days in culture, BMECs typically reached 80–90% confluency.

### Preparation of in vitro BBB models

BMECs (4×10^4^ cells/well) were seeded on the inside of the fibronectin-collagen IV (0.1 and 0.5 mg/mL, respectively)-coated polyester membrane (0.33 cm^2^, 0.4 µm pore size) of a Transwell®-Clear insert (Costar, Corning, NY) placed in the well of a 24-well culture plate (Costar). Cells were cultured in DMEM/F-12 supplemented with 20% PDS, 100 units/mL penicillin, 100 µg/mL streptomycin, 50 µg/mL gentamicin, 2 mM GlutaMAX™-I, 1 ng/mL bFGF and 500 nM hydrocortisone (Sigma) at 37°C with a humidified atmosphere of 5% CO_2_/95% air until the BMEC monolayers reached confluency (3 days). To check the integrity of the BMEC monolayers, transendothelial electrical resistance was measured before the experiments using an EVOM voltohmeter equipped with STX-2 electrode (World Precision Instruments, Sarasota, FL). The TEER of cell-free Transwell®-Clear inserts were subtracted from the obtained values.

### Endoglycosidase F1 treatment

Virus was treated with endoglycosidase F1 (eF1; Calbiochem®, EMD Biosciences, La Jolla, CA) to cleave high mannose oligosaccharides. HIV-1 (10 µg) was incubated for 24 hr at 37°C in 50 µL of phosphate buffer (pH 5.5) containing 0.5 mM phenylmethanesulfonyl fluoride (Sigma) and 2000 mU/mL eF1. After the incubation period, sample was diluted with phosphate buffer (pH 7.4). To remove eF1 (MW 32,000), the sample was filtered by Microcon® YM-100 (Millipore, Bedford, MA) with the 100K cut-off membrane. The virus retained on the membrane was used for radioactive labeling as described above. The iodinated virus was purified by filtration on Sepharose 4B-200 (Sigma) columns. Control HIV-1 was incubated in the same manner without eF1.

### Transendothelial transport of ^131^I-HIV-1

For the transport experiments, the medium was removed and BMECs were washed with physiological buffer containing 1% BSA (141 mM NaCl, 4.0 mM KCl, 2.8 mM CaCl_2_, 1.0 mM MgSO_4_, 1.0 mM NaH_2_PO_4_, 10 mM HEPES, 10 mM D-glucose and 1% BSA, pH 7.4). The physiological buffer containing 1% BSA was added to the outside (abluminal chamber; 0.6 mL) of the Transwell® insert. To initiate the influx of ^131^I-HIV-1 experiments, ^131^I-HIV-1 (3×10^6^ cpm/mL) was loaded on the luminal chamber. The side opposite to that to which ^131^I-HIV-1 was loaded is the collecting chamber. Samples were removed from the collecting chamber at 15, 30, 60 and 90 min and immediately replaced with an equal volume of fresh 1% BSA/physiological buffer. The sampling volume from the abluminal chamber was 0.5 mL. All samples were mixed with 30% trichloroacetic acid (TCA; final concentration 15%) and centrifuged at 5,400×g for 15 min at 4°C. Radioactivity in the TCA precipitate was determined in a gamma counter. The permeability coefficient and clearance of TCA-precipitable ^131^I-HIV-1 were calculated according to the method described by Dehouck et al. [32]. Clearance was expressed as microliters (uL) of radioactive tracer diffusing from the luminal to abluminal (influx) chamber and was calculated from the initial level of radioactivity in the loading chamber and final level of radioactivity in the collecting chamber: Clearance (uL)  =  [C]_C_ × V_C_/[C]_L_, where [C]_L_ is the initial radioactivity in a uliter of loading chamber (in cpm/uL), [C]_C_ is the radioactivity in a uL of collecting chamber (in cpm/uL), and V_C_ is the volume of collecting chamber (in uL). During a 90-min period of the experiment, the clearance volume increased linearly with time. The volume cleared was plotted versus time, and the slope was estimated by linear regression analysis. The slope of clearance curves for the BMEC monolayer plus Transwell® membrane was denoted by PS_app_, where PS is the permeability × surface area product (in uL/min). The slope of the clearance curve with a Transwell® membrane without BMECs was denoted by PS_membrane_. The real PS value for the BMEC monolayer [33] was calculated from 1/PS_app_  = 1/PS_membrane_ +1/PS_e_. The PS_e_ values were divided by the surface area of the Transwell® inserts (0.33 cm^2^) to generate the endothelial permeability coefficient (P_e_, in cm/min).

The effects of mannose, mannan, and mannose-6-phosphate, (all purchased from Sigma) on ^131^I-HIV-1 transport were determined by adding various concentrations of mannose (1–50 mM), mannan (1–5 mg/mL), and mannose-6-phosphate (1–10 mM) to the loading chamber with ^131^I-HIV-1. Effects of 8-bromo-cAMP (0.1–1 mM, Sigma) and forskolin (1–30 mM, Sigma) on ^131^I-HIV-1 transport was determined by pretreatment of BMECs for 30 min prior to the transport experiment.

### Uptake of ^131^I-HIV-1 by BMECs

The uptake of ^131^I-HIV-1 by BMECs was measured based on the method for microvessels reported previously (Banks et al., 2004) with a minor modification. BMECs were cultured in 24 well culture plates for 3–4 days and washed three times with 1% BSA/physiological buffer. BMECs were incubated with 200 mL of 1% BSA/physiological buffer containing ^131^I-HIV-1 (3×10^5^ cpm) and ^125^I-albumin (1×10^6^ cpm) for 15 min at 37°C. The incubation supernatants were removed, and then cells were washed four times with ice-cold physiological buffer. An acid wash technique was used to remove the labeled virus binding to cell surface and to evaluate the amounts of the labeled virus internalized into BMECs. The cells were incubated with ice-cold acid wash buffer (28 mM sodium acetate, 120 mM NaCl and 20 mM sodium barbital, pH 3.0) for 6 min at 4°C. The buffer was removed and the cells were subsequently washed again. Acid washed BMECs were lysed with 150 mL of 1M NaOH. Aliquots (50 mL) of cell lysate were measured cellular protein by BCA Protein Assay Reagent (Pierce, Rockford, IL). To determine the TCA-precipitable radioactivity in the cell lysate (intracellular ^131^I-HIV-1) and incubation buffer (surface-bound ^131^I-HIV-1), aliquots of cell lysate (50 uL) and incubation buffer (100 microL) were mixed with 30% TCA (final concentration 10%) and centrifuged at 5,400×g for 15 min at 4°C. Radioactivity in the TCA precipitate was determined in a gamma counter. The uptake of TCA-precipitable radiolabeled protein by BMECs was expressed as the cell/medium ratio (radiolabeled protein amounts in the cells (in cpm/mg protein)/radiolabeled protein concentration in the incubation buffer (in cpm/uL)).

Various concentrations of mannose (1–30 mM), mannan (1–5 mg/mL), mannose-6-phosphate (0.1–10 mM), N-acetyl-D-glucosamine (GlcNAc, 0.1–50 mM) was incubated with ^131^I-HIV-1. Effects of BAPTA (intracellular Ca^2+^ chelator, 5–20 µM), A23187 (calcium ionophore, 1–10 µM), and verapamil (calcium channel blocker, 20 uM) on ^131^I-HIV-1 uptake were determined by pretreatment of BMECs for 30 min prior to the uptake experiment. Wheat germ agglutinin (WGA, 1 µg/mL) and protamine sulfate (1 mg/mL) were incubated with ^131^I-HIV-1 and ^125^I-albumin in the presence of various concentrations of mannan (1–5 mg/mL). All test compounds were obtained from Sigma.

### Statistics

Statistical analysis was performed with the use of the Prism 5.0 program (GraphPad Software, Inc., San Diego, CA). Means are shown with their standard error terms. Regression lines were calculated by the least-squares method and are reported with their correlation coefficient, *r*, *n*, and *p* values. Regression lines were compared statistically with the Prism 5.0 program. One-way analysis of variance (ANOVA) with Newman-Keuls multiple comparison test and Dunnett's multiple comparison test were calculated by using Prism 5.0 Means are reported with their standard error terms and *n*. Two means were compared by *t* test analysis.

## Results

**Figure 1:**
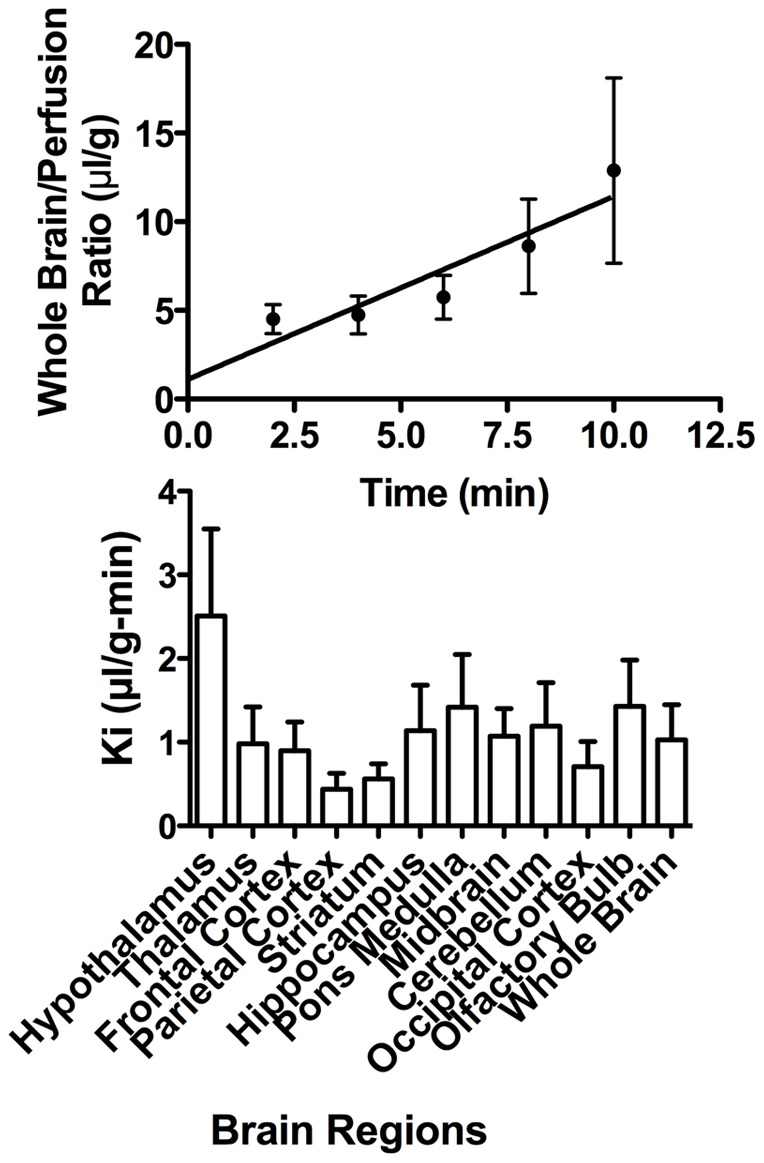
Uptake of HIV radioactively labeled with iodine (I-HIV) by brain. Upper panel shows statistically significant relation between brain/perfusion ratios and time for whole brain with a unidirectional influx rate (Ki) of 1.03 +/− 0.42 ul/g-min. Lower panel shows Ki for brain regions for which ANOVA showed no statistical differences.

Figure 1 (upper panel) shows the results for the uptake as determined with the brain perfusion method. The Ki was 1.03 +/− 0.42 ul/g-min and the Vi was 1.11 +/− 2.77 ul/g for whole brain. The Ki for brain regions is shown in figure 1 (lower panel) and ranged from 2.51 +/− 1.04 ul/g-min for hypothalamus to 0.44 +/−0.19 ul/g-min for parietal cortex. ANOVA found no statistical difference among the Ki values. The Vi ranged from (−1.19) +/− 6.71 ul/g for hypothalamus to 3.98 +/− 3.6 ul/g for olfactory bulb (data not shown); ANOVA found no statistical differences among the Vi values. This uniformity of uptake is consistent with widespread distribution of binding sites at the BBB.

**Figure 2:**
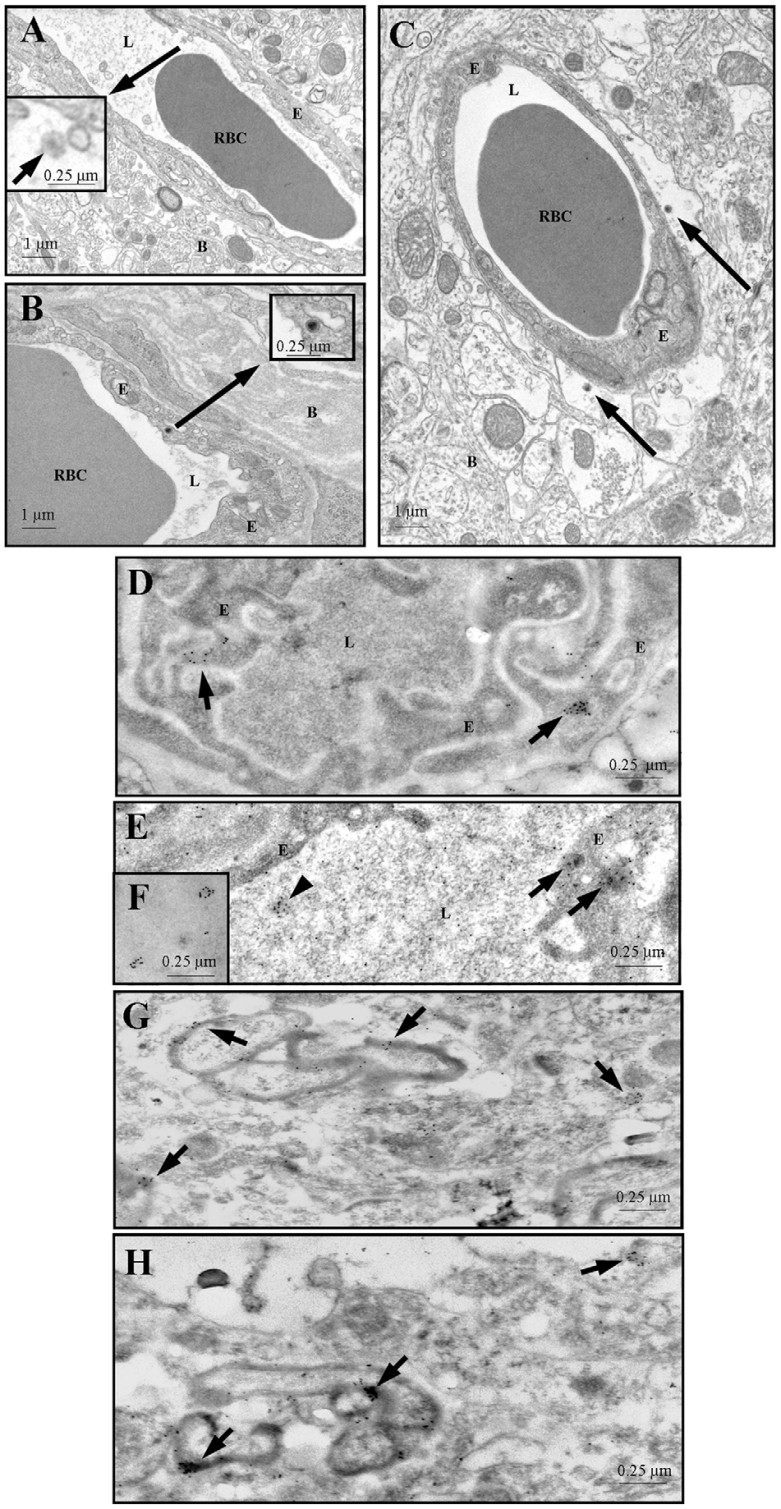
Panels A and B: HIV particles (arrows) in the lumen (panel A) and in an endothelial cell (panel B) of blood vessels in mouse cerebral cortex. The boxed regions show higher magnification images. Panel C: HIV (arrows) have traversed the endothelium of a blood vessel in mouse brain cerebral cortex and gained access the perivascular space. Panels D–F: Colloidal gold labeled HIV in the lumen (arrowhead in panel E, and panel F) and in endothelial cells (arrows in panels D and E) of mouse cerebral cortex capillaries. Panels G and H: HIV associated with myelin sheaths (arrows) of nerve axons in mouse cerebral cortex. Thin sections were incubated with HIV-1SF2 gp120 antiserum diluted 1:100 and followed, after washing, by incubation in protein A-10 nm gold diluted 1∶100. B – brain parenchyma, E – endothelium, L – capillary lumen, RBC – red blood cell.

Ultrastructural examination revealed virus particles in the capillaries and endothelial cells of the cerebral cortex (Figure 2, panels A and B) and in the brain parenchyma (Figure 2, panel C) 5 min after its injection into the jugular vein, indicating that virus had traversed the blood-brain barrier. HIV particles are 150 nm in diameter and contain cone-shaped cores in tissue sections [34]. These observations were confirmed by immuno-gold localization on thin sections of PFA-fixed but non-osmicated tissue embedded in LR White using antibodies to HIV-1's gp120 coat glycoprotein and protein A-10 nm colloidal gold (Figure 2, panels D–H. The abundance of labeled HIV particles decreased with distance from capillaries as expected from the relatively short perfusion time in these experiments. Virus is preferentially associated with the myelin sheaths of myelinated nerves in the brain parenchyma (Figure 2, panels G–H).

**Figure 3:**
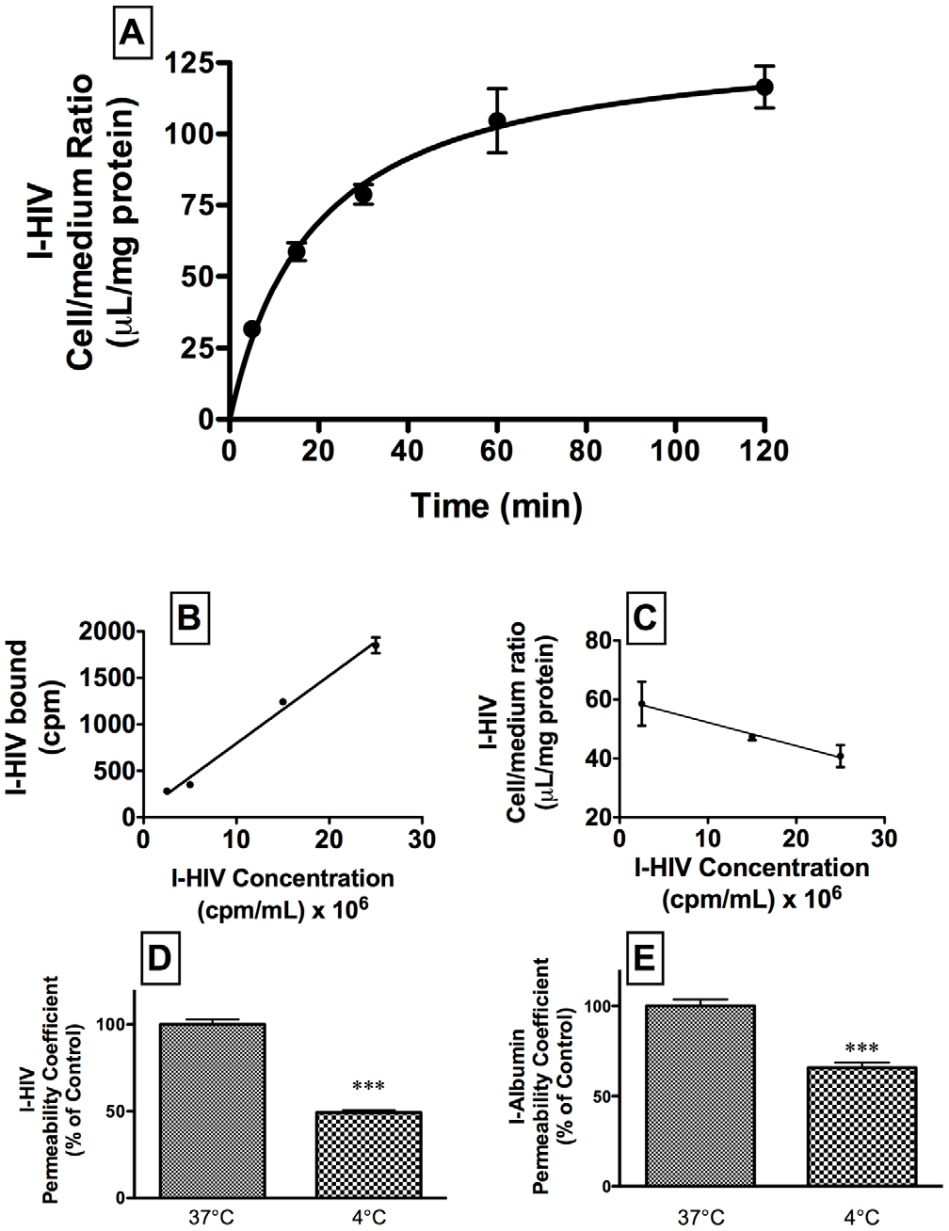
Panel A shows kinetics of uptake of radioactively labeled HIV-1 (I-HIV) over time by BMEC. Panels B and C show linearity with increases concentrations of I-HIV. Temperature decreased permeation of I-HIV (panel D) and of I-Albumin (panel E) in BMEC.

Uptake of I-HIV by BMEC during a 120 min study is shown in panel A of figure 3 (n = 4/time point). The results were fitted to a one-site hyperbolic model with a Bmax of 135 +/− 9 ul/g and a Kd of 19.1 +/− 4.0 min (r = 0.935, n = 20). The relation was linear for the first 30 min (r = 0.942, n = 12, p<0.001) and subsequent tests for BMEC uptake were done at 30 min. Increasing concentrations of I-HIV resulted in a linear increase in binding to the cell membranes (Figure 3, panel B) and a decreasing cell/medium ratio (figure 3, panel C) consistent with I-HIV-1 binding to a receptor site on BECs. The effects of temperature on I-HIV (t = 16.1, df = 10, p<0.001) and I-Alb (t = 7.3, df = 10, p<0.0001) on transport across the BMEC monolayers are shown in figure 3 panels D and E, respectively (n = 6/group). Raw values at 37°C were (9.1 +/− 0.6)10^−6^ cm/sec for I-HIV and (7.4 +/− 1.9)10^−6^ cm/sec for I-Alb and at 4°C were (4.5 +/− 0.2)10^−6^ cm/sec for I-HIV and (4.6 +/− 1.0)10^−6^ cm/sec for I-Alb. Thus, in comparison to 37°C, at 4°C, transport of I-HIV was inhibited by about half and transport of I-Alb was inhibited by about one-third.

**Figure 4:**
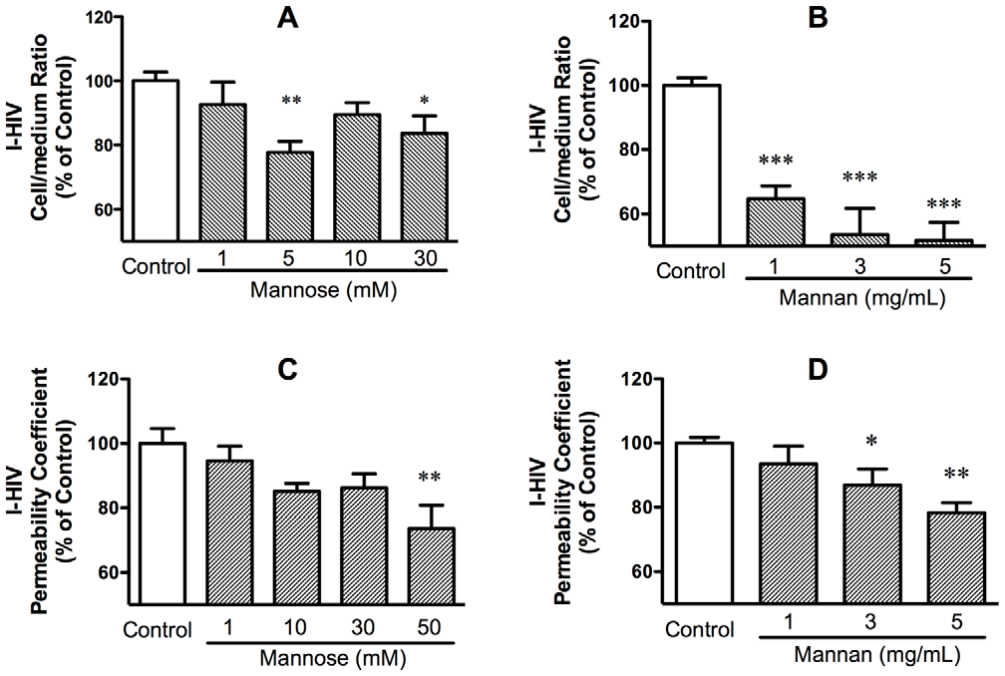
Uptake (panels A and B) and transport (panels C and D) were inhibited by mannose (panels A and C) and by mannan (panels B and D).

Mannose and mannan, blockers of M6PR and other mannose binding sites, were effective blockers of I-HIV uptake and transport. Figure 4, panel A shows that mannose inhibited I-HIV uptake [F(4,28) = 4.39, p<0.01] and figure 4, panel C shows that mannose inhibited I-HIV transport [F(4,40) = 4.04, p<0.01]. Figure 4, panel B shows that mannan inhibited I-HIV uptake [F(3,33) = 20.6, p<0.01] and Figure 4, panel D shows that mannan inhibited I-HIV transport [F(3,23) = 7.23, p<0.01]. These results show that a mannose binding site such as M6PR is involved in HIV-1 transport across the BBB.

**Figure 5:**
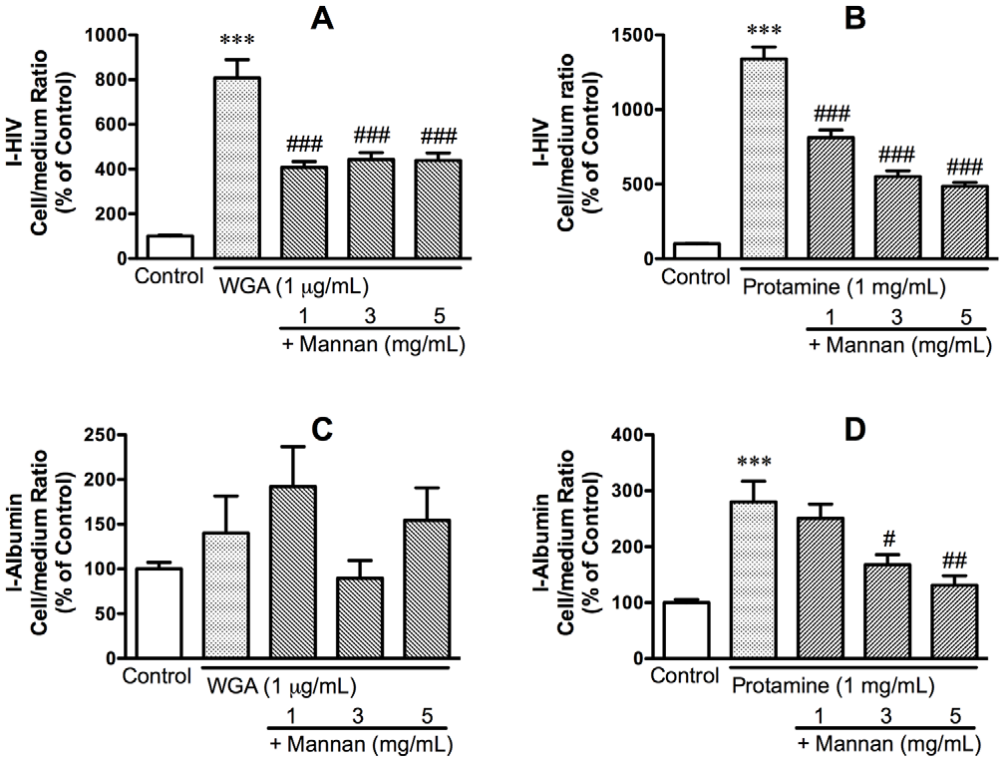
Mannan blocked the increase in I-HIV uptake induced by WGA (panel A) and by protamine (panel B) in BMEC cells. Neither WGA nor WGA + mannan had an effect on I-Albumin uptake (panel C). Protamine increased uptake of I-Albumin by BMEC although much less robustly than the uptake of I-HIV; mannan blocked this effect, although at higher doses than for the protamine/HIV-1 effect (panel D).

WGA and protamine sulfate greatly increase the permeation of HIV-1 across the BBB and it is assumed that they potentiate the endogenous mechanism by which HIV-1 crosses the BBB. Mannan blocked both the WGA-induced and the protamine-induced increases in I-HIV uptake. Figure 5, panel A shows the results for WGA on I-HIV uptake: F(4,46) = 30.8, p<0.001. Tukey's comparison test showed that WGA significantly increased I-HIV uptake by 8 fold in comparison to control (p<0.01) and that mannan at each concentration reduced I-HIV uptake in comparison to the WGA only group (each comparison at p<0.01). Neither WGA nor WGA + mannan had an effect on I-Alb uptake (Figure 5, panel C). Figure 5, panel B shows the results for protamine sulfate on I-HIV-1 uptake: F(4,38) = 88.6, p<0.01. Tukey's comparison test showed that protamine increased I-HIV uptake by over 13 fold in comparison to control (p<0.01) and that mannan at each concentration reduced I-HIV uptake in comparison to the protamine only group (each comparison at p<0.01). Protamine also had an effect on I-Alb uptake (Figure 5, panel D): F(4,38) = 10.4, p<0.01. Tukey's comparison test showed that protamine increased I-Alb uptake by almost 3 fold in comparison to control (p<0.01) and that mannan at the 3 (p<0.05) and 5 (p<0.01) mg/ml concentrations reduced I-Alb uptake in comparison to the protamine only group. For the protamine experiments, the control value for I-HIV was 24.4 +/− 3.9 ul/mg-protein and for I-Alb was 3.3 +/− 0.7 ul/mg-protein and the protamine-stimulated values were 310 +/− 48.9 ul/mg-protein for I-HIV and 10 +/− 2.9 ul/mg-protein for I-Alb. GlcNAc had no effect on I-HIV uptake. Overall, these results show that the WGA and protamine sulfate enhanced permeation of HIV-1 across the BBB is mediated through mannose binding sites.

**Figure 6:**
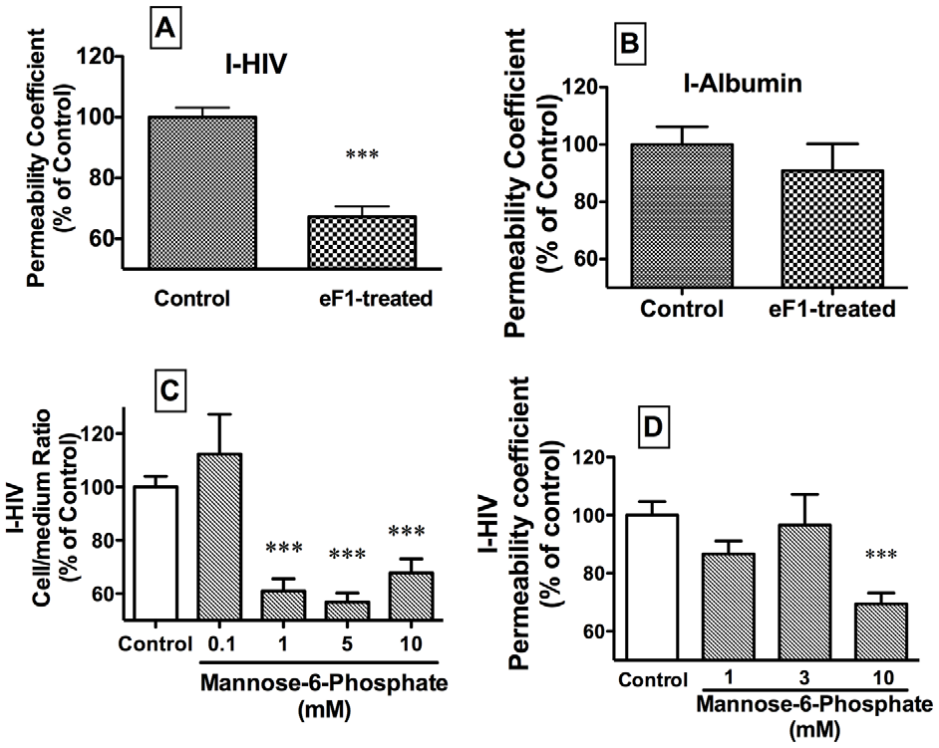
Panel A: Cleavage of high mannose oligosaccharides from HIV with endoglycosidase F1 (eF1) reduced the transport of I-HIV across BMEC. Panel B: The internal control of I-Albumin was unaffected. Mannose-6 phosphate, a ligand for and competitive inhibitor of the mannose-6 phosphate receptor, inhibited the uptake (panel C) and permeation (panel D) of I-HIV across BMECs.

Figure 6 (panel A) shows that treating HIV-1 with endoglycosidase F1, an enzyme which cleaves high mannose oligosaccharides, reduced its transport by about 1/3 (t = 7.08, df = 18, p<0.001). Neither permeation of the internal control I-Alb (Figure 6, panel B) nor TEER (data not shown) were affected. This shows that high mannose oligosaccharides are key to the transport of HIV-1 across the BBB.

Whereas the above shows that mannose binding sites are involved in HIV-1 transport, mannose-6-phosphate was used to determine whether M6PR was specifically involved. Mannose-6-phosphate inhibited both I-HIV uptake and transport. Uptake (Figure 6, panel C) was inhibited as shown by ANOVA [F(4,22) = 17.3, p<0.001, n = 3–6/group] and Dunnets showed that concentrations of 1, 5, and 10 mM, but not 0.1 mM, inhibited uptake (p<0.001). Transport (Figure 6, panel D) was inhibited by as shown by ANOVA [F(3,38) = 7.4, p<0.001, n = 6–12/group] and Dunnets showed that the concentration of 10 mM (p<0.001), but not of 1 or 3 mM, inhibited transport. These results show that M6PR was specifically involved in HIV-1 transport across the BBB.

**Figure 7:**
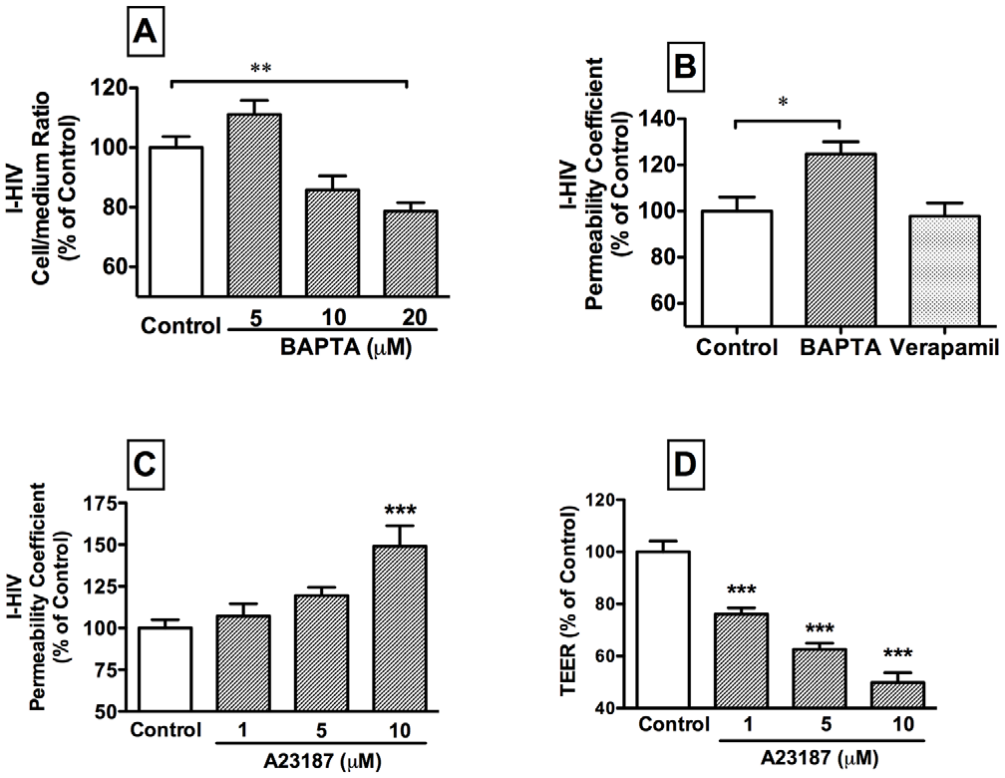
The intracellular calcium chelator BAPTA decreased cell retention of I-HIV at 20 uM (panel A) and increased transport rate of I-HIV at 10 uM (panel B). This is consistent with an increased rate of transport across the BMEC. The calcium channel blocker verapamil had no affect on transport. The calcium ionophore A23187 increased I-HIV permeability (panel C) and decreased TEER (panel D) in BMEC.

We tested the importance of calcium on HIV-1 transport with a calcium ionophore (A23187), a calcium chelator (BAPTA), and a calcium channel blocker (verapamil). The intracellular calcium chelator BAPTA affected cell uptake of I-HIV as shown in Figure 7, panel A [F(3,35) = 12.6, p<0.001; n = 9/group]. Dunnett's multiple comparison test showed that only 20 uM of BAPTA produced a statistically significant effect (p<0.01) in comparison to controls. BAPTA (10 uM) increased transport of I-HIV across monolayers (Figure 7, panel B): t = 3.06, df = 10, n = 6/group, whereas verapamil (20 uM) was without effect. BAPTA had no effect on TEER.

The calcium ionophore A23187 increased I-HIV transport [F(3,29) = 7.3, p<0.001, n = 6–9/group] at the 10 uM dose (p<0.001 by Dunnett's); Figure 7, panel C. This compound also decreased TEER [F(3,29) = 41.6, p<0.001, n = 6–9/group)] at each of the three doses tested (each with p<0.001 by Dunnett's): Figure 7, panel D.

**Figure 8:**
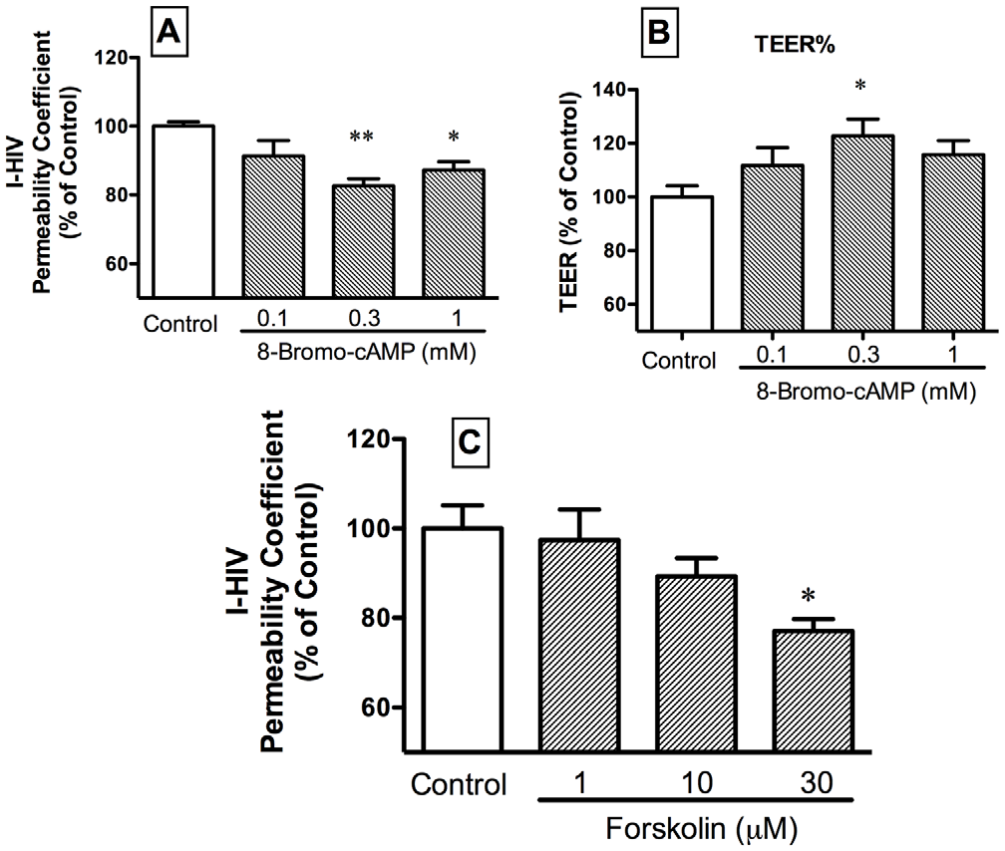
The cyclic AMP analog 8-bromo-cAMP decreased transport of HIV-1 across BMEC (panel A) and at the 0.3 mM dose increased TEER (panel B). The adenylate cylcase activator forskolin decreased transport of HIV-1 across BMEC (panel C).

We tested the importance of cAMP in HIV-1 transport by using a cAMP analog (8-bromo-cAMP) and a adenylate cyclase activator (forskolin). Figure 8, panel A shows that the cAMP analog 8-bromo-cAMP partially inhibited I-HIV transport across BMEC: F(3,21) = 5.77, p<0.01 (n = 5–6/group). TEER was increased at the intermediate dose by 8-bromo-cAMP (Figure 8, panel B): F(3,23) = 2.84, p = 0.06 (n = 6/group). The adenylate cyclase activator forskolin (Figure 8, panel C), also inhibited I-HIV transport at its high dose: F(3,17) = 3.41, p<0.05 (n = 3–6/group).

## Discussion

These studies show that HIV-1 uses the mannose-6 phosphate receptor to cross the BBB as free virus. In brief, we used in vivo pharmacokinetic methods, ultrastructural studies, and an in vitro polarized monolayer model of the BBB in our studies.

We used as a basis for these studies key findings and models previously used to study HIV-1 transport across the BBB. We used a virus that was rendered non-infective by aldrithiol-2, a substance that binds the zinc fingers of HIV-1 RNA. This treatment leaves the viral coat unaltered and indeed was developed for use as a vaccine [21], [22]. Being non-infective conveys the advantage that any virus we detect is known to be material which we injected into the circulation; this is an important point as otherwise virus found in brain could be assumed to have arisen from sites of infection rather than immediately from blood. We previously showed that the species barrier for HIV-1 does not extend to BBB penetration; that is, HIV-1 is just as rapidly and efficiently taken up by mouse brain endothelial cells as by human brain endothelial cells [35].

We first confirmed and extended previous in vivo studies showing that free HIV-1 can cross the BBB of the mouse [4]. In previous work, we used 4B-200 Sepharose chromatography to demonstrate that radioactivity taken up by brain represents intact virus and not viral coat proteins or degradation products [8]. Free virus also crosses much faster than its surface coat protein gp120. Here, we found that uptake of HIV-1 by the brain was rapid and occurred throughout the CNS (Figure 1), consistent with the widespread distribution of M6PR within the CNS [36]. Based on specific activity of our I-HIV and published estimates of protein molecules per virion, we roughly estimate that we infused 10^5–6^ virions/ml [37]. This is well within the range of free virus concentrations in patients with AIDS which typically is between 10^3^ and 10^8^ virions/ml [38], [39]. The rate of 1 ul/g-min is as fast as some small peptides that are transported by saturable systems and about 500 times faster than the rate at which vascular markers such as albumin leak into the brain.

Ultrastructural studies confirmed both the BBB penetration of HIV-1 free virus and its rapid uptake (Figure 2). Five minutes after iv injection of HIV-1, we found virus in the lumen, internalized in brain endothelial cells, and in brain tissue. These results were seen both with standard transmission electron microscopy and with immuno-Gold staining. HIV-1 was located within the cytoplasm of the brain endothelial cells rather than associated with intercellular structures. This is consistent with HIV-1 crossing the BBB by the transcytotic route, although passage by the paracellular route as well was not ruled out. The dissociation of TEER and HIV-1 permeability in the dose-response curves for the calcium ionophore A23187 (Figure 7) and the cAMP analog 8-bromo-cAMP (Figure 8) also argues against HIV-1 crossing exclusively by way of the paracellular route. However, calcium is also important in tight junction regulation and it is possible that paracellular transport of HIV-1 was enhanced as well [40]. Immunogold staining showed that HIV-like immunoactivity had a propensity for myelinated neurons (Figure 2 G–H). Loss of myelin is a hallmark of the HIV-1 infected brain and is thought to be mediated through viral proteins, cytokines, neurotoxins, and antibodies or enzymes directed against myelin [41], [42], [43]. The association of HIV-1 with myelin so immediately after its crossing reinforces these ideas and suggests that viral effects on myelin could be direct and occur early.

We then used the in vitro monolayer model of the BBB to further characterize and identify the transporter used by HIV-1 to cross the BBB. We used primary cultures of mouse brain microvascular endothelial cells (BMECs) grown as monolayers on transwell inserts [44]. The in vivo BBB is polarized; that is, the lipids, proteins, and transporters present on its luminal (blood side) surface differ from those on its abluminal (brain side) surface. The monolayer model used here polarizes after the fashion of the in vivo BBB with the cell membrane facing into the buffer expressing luminal properties and the cell membrane attached to the collagen expressing abluminal properties. Thus, these monolayers are ideal for studying BBB transport. Previous work has shown that transport of HIV-1 across BMECs is a multi-stage process with different properties for adherence, internalization, and transport [8], [45]. We, therefore, studied uptake (cell/medium ratios) and permeation (permeability coefficients). Uptake involves adherence and internalization of HIV-1 by brain endothelial cells, whereas permeation reflects the complete transport of HIV-1 across the brain endothelial cell from the donor (luminal) chamber into the acceptor (abluminal) chamber. As expected, uptake was time dependent and transport was temperature sensitive (Figure 3), consistent with the vesicular- and energy-dependent processes previously shown for HIV-1 transport across the BBB. At 37°C, HIV-1 transport was 23% faster than that of I-Alb, despite the much greater size of HIV-1. This demonstrates a selectivity to HIV-1 transport across the monolayer as compared to I-Alb.

A series of experiments showed that HIV-1 transport was dependent in part on binding to the mannose-6-phosphate receptor (M6PR). Mannose, the mannose polymer mannan, and mannose-6 phosphate each inhibited the uptake and transport of HIV-1, demonstrating that transport was dependent on a mannose binding site (Figure 4 and 6). WGA and protamine sulfate, agents known to enhance the in vivo uptake of HIV-1 and gp120 [4], [6], [7], were demonstrated here to enhance uptake of HIV-1 in our in vitro model. It has been assumed that these substances are enhancing the endogenous transport mechanism used by HIV-1 to cross the BBB. Finding that these enhanced uptakes were blocked by mannan was consistent with this assumption, demonstrating that WGA and protamine sulfate act at least in part through a mannose binding site (Figure 5). The importance of the mannose binding site was further illustrated by treating HIV-1 with eF1, an enzyme that cleaves high mannose oligosacharrides. This decreased the ability of HIV-1 to cross the monolayer, demonstrating the importance of these sugars in transport (Figure 6). Finally, we showed that HIV-1 uptake and transport were inhibited by mannose-6 phosphate, thus demonstrating the mannose binding site was M6PR.

Overall, these studies show the importance of mannose binding sites, especially the M6PR, in HIV-1 transport across brain endothelial cells. They do not show that M6PR is exclusively used by HIV-1 nor do they show that all of the mannose sensitive transport is due to M6PR. They do show that M6PR is a major transporter used by HIV-1.

Two versions of M6PR are known: a cation-dependent (CD-M6PR) and a cation-independent (CI-M6PR) version [46]. The work with the calcium chelator BAPTA and the calcium ionophore A23187 shows an affect of calcium and so strongly suggests that the CD-M6PR is involved in HIV-1 transport. The smaller (46 kDa) CD-M6PR has one binding site for mannose-6-phosphate and the larger (300 kDa) CI-M6PR has three binding sites for mannose-6 phosphate, one of which also binds GlcNAc [47]. We showed that GlcNAc had no effect on HIV-1 uptake, suggesting that the CI-M6PR is not involved, although it may be that GlcNAc binding does not sterically interfere with HIV-1 binding. The intracellular calcium chelator BAPTA decreased uptake but increased transport of HIV-1 across the BMEC monolayer (Figure 7). These seemingly contradictory findings can be reconciled by the conclusion that BAPTA enhanced transit time of HIV-1 across the monolayer. The calcium ionophore A23187 had the opposite effect of calcium chelation in that it enhanced HIV-1 transport; this could be a result of effects on tight junctions or on transcytotic processes. Verapamil, a calcium channel blocker, was without effect, suggesting that calcium channels are not involved in HIV-1 transport. Overall, these effects suggest that HIV-1 uses the calcium dependent M6PR to cross the BBB.

Previous work has shown that cAMP inhibits the activity of IGF-2, an action mediated through M6PR [48], [49]. We postulated that this down regulation could be evidence that cAMP induces the internalization of the M6PR. If so, the cAMP would be predicted to enhance HIV-1 transport. A role for cAMP in HIV-1 transport across the brain endothelial cell was shown in that both the cAMP analog 8-bromo-cAMP and the cAMP activator forskolin both enhanced transport (Figure 8). Thus, cAMP is an important part of the intracellular machinery controlling HIV-1 transport across the BBB.

In conclusion, we showed with both pharmacokinetic and ultrastructural studies that intravenous HIV-1 rapidly crosses the BBB. We used an in vitro model of the BBB to show that the mannose-6-phosphate receptor mediates this transport.
